# Supplementary material for: Healthcare professionals’ perception and COVID-19 vaccination attitudes in North-Western Ghana: A multi-center analysis
Source: PLoS One. 2024 Feb 22;19(2):e0298810. doi: 10.1371/journal.pone.0298810 (PMC10883535; doi:10.1371/journal.pone.0298810)
Supplement: S1 Questionnaire — (DOCX) [file pone.0298810.s001.docx]

**QUESTIONNAIRE ON PERCEPTION AND ATTITUDES TOWARDS COVID-19**

**VACCINATION**

| **S/N** | **VARIABLES** | **Response Categories** |  |
| --- | --- | --- | --- |
| **Form Identification Details** | | |  |
|  | Date of interview |  |  |
|  | Name of research Assistant |  |  |
|  | Form Number |  |  |
| **SECTION A: Sociodemographic characteristics of caregivers and Children** | | |  |
|  | Respondents Sex | 1. Male 2. Female |  |
|  | Respondents’ Age (Yrs) |  |  |
|  | Respondents’ Marital status | 1. Single 2. Married 3. Divorced/separated 4. Widowed 5. Co-Habitating |  |
|  | Religion | 1. Christianity 2. Islam 3. Traditional |  |
|  | Ethnic background | - - - 1. Waala       2. Daagba       3. Akan       4. Sissala       5. Others specify………. |  |
|  | What is your Education Qualification | 1. Certificate 2. Diploma 3. Degree 4. Masters/PhD |  |
|  | What is your profession | 1. Nurse 2. Physicians 3. Biomedical Scientist 4. Pharmacist/ technician 5. Others specify (…..) |  |
|  | Sub-Municipal |  |  |
|  | Type of work setting | - - - 1. Private       2. Government       3. Quasi facility |  |
|  | Type of facility | CHPS  Clinic  Health center  Polyclinic  Municipal hospital  Regional hospital |  |
|  | Name of current facility |  |  |
|  | Duration in active service (Years) |  |  |
| **SECTION B: Perception about COVID-19 Vaccination** | | |  |
|  | Do you know anyone infected with COVID-19? | 1. Yes 2. No |  |
|  | Do you think you have come in contact with COVID-19? | 1. Yes 2. No |  |
|  | Are you afraid of getting infected? | 1. Yes 2. No |  |
|  | Do you think COVID-19 is real | Yes  No |  |
|  | Do you have protection measures in your work place? | 1. Yes 2. No |  |
|  | Which of the following Vaccines do you think is safe? | 1. Moderna Spikevax 2. Oxford Astra-Zeneca vaccines 3. Gamaleya Sputnik V 4. Pfizer/BioNtech Comirnaty 5. Janssen (Johnson & Johnson) 6. Serum institute of India Covishield (Oxford /Astra-Zeneca formulation 7. All 8. None |  |
|  | COVID-19 vaccination will prevent spread of infection to patients | 1. Disagreed 2. Neutral 3. Agreed |  |
|  | COVID-19 vaccination will prevent spread of infection among hospital workers | 1. Disagreed 2. Neutral 3. Agreed |  |
|  | The vaccines produced by the pharmaceutical companies are safe and effective vaccines | 1. Disagreed 2. Neutral 3. Agreed |  |
|  | Do you believe that the vaccines produce  an immune response against COVID-19? | 1. Disagreed 2. Neutral 3. Agreed |  |
| **SECTION D: Attitude** | | |  |
|  | Ever had a COVID-19 Infection? | - 1. Yes   2. No |  |
|  | Have you received the COVID-19 Vaccination? | Yes  No- (Skip to Question 18) |  |
|  | If Yes, Which of the Vaccines? | 1. Moderna Spikevax 2. Oxford Astra-Zeneca vaccines 3. Gamaleya Sputnik V 4. Pfizer/BioNtech Comirnaty 5. Janssen (Johnson & Johnson) 6. Serum institute of India Covishield (Oxford /Astra-Zeneca formulation |  |
|  | If Yes, Have you completed the COVID-19 vaccination? | 1. Yes (skip to 20) 2. No |  |
|  | If No, Why? | 1. Vaccine not available 2. Side Effect of Initial Shot(s) 3. Others specify……. |  |
|  | If No, Reasons for not taking COVID-19 vaccines | 1. Infertility 2. Severe Allergic Reactions 3. Unknown long time effects | |
|  | If no, are you planning to receive the vaccine in the near future? | 1. Yes 2. No | |
|  | What alternative preventive measures did you use to prevent COVID-19 | 1. Traditional medicine 2. Praying 3. Physical exercise 4. Adhering to COVID prevention protocols | |
|  | Should people with chronic and severe diseases get priority for COVID-19 vaccination? | 1. Yes 2. No | |
|  | Should HCWs get priority in COVID-19 vaccination? | 1. Yes 2. No | |
|  | To protect the public, HCWs should follow government guidelines about vaccines | 1. Yes 2. No | |
