# Supplementary material for: Healthcare professionals’ perception and COVID-19 vaccination attitudes in North-Western Ghana: A multi-center analysis
Source: PLoS One. 2024 Feb 22;19(2):e0298810. doi: 10.1371/journal.pone.0298810 (PMC10883535; doi:10.1371/journal.pone.0298810)
Supplement: Attachment — Submitted filename: reviewers comments.pdf [file pone.0298810.s003.pdf]

I am pleased to review and provide my opinion on the topic titled **'Perception and COVID-19 Vaccination Attitudes Among Healthcare Professionals: A Multi-Centre Cross-Sectional Hierarchical Logit Analysis in North-Western Ghana**.'

Overall, the article provides a comprehensive analysis of healthcare professionals' perceptions and attitudes toward COVID-19 vaccination in the Wa Municipality, Upper West Region, Ghana. However, there are some areas that could be improved for clarity and precision:

- The current title is broad. Consider enhancing specificity and conciseness to better reflect the study's main focus. A suggested revised title is: 'Healthcare Professionals' Perception and COVID-19 Vaccination Attitudes in North-Western Ghana: A Multi-Centre Analysis.'
- The abstract should provide a concise summary of the study, including the objectives, methods, results, and conclusion. It currently lacks a clear statement of the study's purpose.
- The introduction should clearly state the problem, the significance of the study, and the research questions or objectives.
- It's important to include a literature review to provide context and highlight the gap in knowledge that this study aims to fill.
- I would suggest you seen how to incorporate the following systematic review https://journals.plos.org/plosone/article?id=10.1371/journal.pone.0289295
- Provide context for the problem: What is the actual issue in the Wa Municipality, Upper West Region?
- Clarify the sampling method used to select the 420 health professionals initially sampled.
- Offer additional information about the survey tool, including its development or adaptation. However, the reliability (Cronbach's alpha = 0.67, α = 0.64, and α = 0.60) and validity appear questionable. Was the correct data collected?"
- Some of the tables have a lot of information; consider breaking them down for easier interpretation.
- Use clear and concise language in presenting the results to enhance readability.
- Provide a more detailed discussion of the results, relating them back to the existing literature.
- You don't need sub-headings the discussion section
- Discuss the implications of the findings for public health and healthcare policy.
- Table 2: Consider providing a clearer title that indicates it is presenting sociodemographic characteristics.
- Table 3: Use more descriptive headers for each column to improve clarity.
- Table 4: Consider organizing the data in a more structured format for better readability.
- Table 5: Consider providing more context for the computed composite scores.
- Check for grammatical errors and ensure consistency in writing style.
- Avoid redundancy and ensure each section adds unique information to the study.
- Provide a concise summary of the key findings and their significance.

- Suggest practical implications and recommendations based on the study.
